# Investigating mating reliability and drone congregation areas on an island in Lake Neusiedl (Austria) for the potential establishment of a mating station for honey bee breeding

**DOI:** 10.5194/aab-68-507-2025

**Published:** 2025-07-31

**Authors:** Thomas E. Sprenger, Cameron Menschhorn, Robert Brodschneider

**Affiliations:** 1 University of Natural Resources and Life Sciences Vienna, Gregor-Mendel-Straße 33, Vienna 1180, Austria; 2 Department of Biology, University of Graz, Universitätsplatz 2, Graz 8010, Austria

## Abstract

The mating behaviour of honey bees (*Apis mellifera*) was studied on the small, beekeeping-free island of the Mörbischer Seefestspiele in Lake Neusiedl, Austria. This island is surrounded by reed (*Phragmites australis*), so the water surface might not be a as good a barrier for reproductive honey bees. Mating reliability was tested by a cordovan mating test on the island. Eleven drone colonies of *Apis mellifera ligustica* var. *cordovan* (cd), which have a recessive gene that colours the chitinous exoskeleton of the homozygous worker bees brown instead of black (wild type, wt), were placed on the island. Seventeen virgin cordovan queens were brought to the island for mating in 2022. After mating, the offspring of these queens were examined for their characteristics, and the proportion of homozygous worker bees with brown chitin was determined. Of the 17 virgin queens brought to the island, 11 were available for examination of their offspring. With the exception of two queens, all of the cd queens from the island had both cordovan and wild-type offspring. Although most of the queen bees had cd offspring predominantly, presumably all of them (except one that exclusively mated with cd drones from the island) mated with drones from the mainland as well. Another single queen showed only cd offspring. The average percentage of cordovan progeny of 63 % appears to be too low for controlled breeding of bees. In May and July 2022, we screened several locations of the island with a balloon and quadrocopter for drone congregation areas. In May, before providing drone colonies to the island, not a single drone was found on the island, whereas in July, a drone congregation area could be located close to the drone colonies. The results suggest that this island is not suitable for establishing a mating station for honey bee breeding programmes. The role of reed in drone and queen flight over water surfaces is discussed.

## 1 Introduction

Honey bees (*Apis mellifera*) can be bred to increase desired qualities improving the colonies' performances. Such characteristics include disease resistance, honey yield, gentleness, or low tendency to swarm (Büchler et al., 2013; Uzunov et al., 2022a). To increase those characteristics, finding suitable animals (queens/drones) is required for further breeding by selecting individuals expressing the desired phenotype (or trait). For producing the next generation of queen bees, these selected breeding animals need to be bred to combine their genetics containing the desired qualities. To combine the valuable genetics, several breeding methods are used in honey bee breeding, such as pure breeding, crossbreeding (Armbruster, 1919), breeding for preservation of endangered strains (De La Rúa et al., 2009), and breeding for resistance to *Varroa destructor* (Rinderer et al., 2010; Uzunov et al., 2022a).

In any of these breeding methods, the greatest challenge is mating control (Plate et al., 2019; Uzunov et al., 2022b). The queen bee mates with several drones during the mating flight (Alber et al., 1955; Ratnieks, 1990; Tarpy et al., 2023) and flies up to 16 km away to find mating partners (Peer and Ferrar, 1956; Ruttner and Ruttner, 1972). Due to the mating biology of the reproductive bees, controlled mating is only possible at isolated mating stations or by means of instrumental insemination (Cobey et al., 2013; Uzunov et al., 2022b). The latter requires skills and equipment beyond those of the average beekeeper. Island mating stations are considered to be the safest. In Austria, despite the presence of islands in various Austrian lakes (Lake Neusiedl, Wörthersee, Lake Constance), only a few attempts have been made to establish island mating stations. Land masses surrounded by water are considered islands even if they have an artificial connection to the mainland (via dam). The lake island of Mörbisch (Lake Neusiedl, Burgenland), approximately 20 ha in size, is surrounded by water. It is connected to the mainland through a 1.8 km long dam, which makes it possible to reach the island by car.

Islands at least 3 km away from the mainland are considered safe enough for the establishment of a mating station (Ruttner, 1979). However, queens fly long distances during their mating flight (Peer and Ferrar, 1956) and even cross extensive areas of water in emergency situations, such as a lack of drones, to mate successfully (Klatt, 1929; Klöpping, 1993). Placing virgin queen bees on an island only, without any drone colonies present, appears therefore unsuitable to test the reliability of a mating station in this case. Queens would cross water surfaces in need of drones for successful mating, and no reliable statement could be made about mating security on the lake island under regular breeding conditions. Experiments in the years 1994 to 1997 on the island of Baltrum suggest that queens cross water areas despite the presence of drones on the island (Neumann et al., 1999). However, it must be taken into account that there were only five drone colonies on the island at the time of the experiments, which, according to current knowledge, is not sufficient to stabilize a drone congregation area (Koeniger et al., 2014). Just like queen bees in need of mating partners do, worker bees have been shown to fly over water surfaces to collect nectar and pollen (Heran and Lindauer, 1963; Ruttner and Ruttner, 1965). Heran and Lindauer (1963), however, reported that many bees lost altitude in flights over mirror-smooth calm water and crashed headlong into the water. The latter supports reports from von Frisch's work at the Wolfgangsee (von Frisch, 1993). Very little is known about the ability of drones to cross water, but it is again Ruttner and Ruttner (1965) that state that drones do not willingly fly over water.

Around the lake island of Mörbisch, a lot of reed (*Phragmites australis*) grows, which could hinder the bees' perception of the water surface and thus not be a true barrier for their flight. It is therefore questionable whether such islands are suitable to be a mating station. For worker bees, structures in the water, such as a bridge of floating boards (Ruttner and Ruttner, 1965), can help them cross water surfaces. Therefore, an investigation under real-world conditions is required to see whether the queens mate exclusively with drones from the island or not.

There are three methods for investigating the reliability of mating stations:

1. The first is the placement of virgin queens at the planned mating apiary without any drones present. If they are not laying fertilized eggs after 30 d, the location is considered safe enough for a breeding site (Drescher, 1965).
2. The second is a paternity analysis of worker bee daughters of queens mated at the mating yard, using molecular markers (Jensen et al., 2005).
3. The final method is using queens and drones having a recessive trait, such as cordovan (Maul, 1972) to judge whether the queens are mated with stock of choice or other drones. Genetically marked queen bees and drones were used by many scientists to test the reliability of mating stations with the cordovan mutant.

The cordovan mutation occurs in some subspecies of honey bees (Tucker, 1986). Cordovan (cd) mutants are bees whose exoskeleton is leathery brown instead of dark gray or black. The cd trait is recessive and therefore only appears in homozygous bees. They are therefore particularly suitable for ascertaining whether intended mating occurred or not: only when cordovan queens are mated with cordovan drones will cordovan workers emerge (Ruttner and Ruttner, 1965). The method with the Cordovan bees is less cost-intensive than the paternity analysis and is therefore used for this study.

The phenotypic analysis of the island-mated queens' offspring allows for determining the degree of undesired influence and to assess the suitability of the island as a mating station. To classify this island as a safe mating station, the queens mated there should show a low degree of influences from the mainland through their progeny. Ruttner (1979) recommends that more than half of the queens mated should exclusively mate with drones from the island. A test of the reliability of the mating station should include the use of at least eight cordovan drone colonies according to Koeniger et al. (2014). If mismating takes place despite the presence of a stable drone congregation population on the lake island, mating of the queens with mainland drones must be assumed. As drones probably avoid water surfaces (Ruttner and Ruttner, 1963, 1965) and as any island-mated queens displaying wild-type-coloured offspring should suggest, queens have flown to the mainland to mate with drones there. This was the case on the North Sea island of Baltrum, where mismating occurred despite the presence of five drone colonies on the island (Neumann et al., 1999). It is questionable whether these would have also occurred if more than five drone colonies had been used.

This raises the question of whether on the investigated island a drone congregation area could be established. Drone congregation areas are locations where usually thousands of drones from many different colonies aggregate and wait for a queen to mate with (Loper et al., 1992; Baudry et al., 1998; Koeniger et al., 2005a, b). Landmarks play an important role in the establishment of drone congregation areas (Galindo-Cardona et al., 2012), and areas are stable over years. Ruttner (1976) analysed the behaviour of drones during the mating season and the characteristics of drone congregation areas. Their experiment was conducted with 250 virgin queens and 3 groups of genetically tagged drones in an Alpine valley near Lunz am See. According to their observations, the animals oriented themselves to terrain marks on the horizon using innate orientation mechanisms. The locations of the drone congregation areas are characterized by their surroundings, small basins or windless areas. In this context, stimulus contrasts and horizon silhouettes are important, and places away from the valley are preferred. Documented flight distances of 7000 m and over mountain ridges of an altitude of up to 1000 m are proof that drones can also cross mountains. In the case of the experiment on the island of Mörbisch, no enclosed terrain or windless areas are available. The drones hence have no orientation markers besides trees and buildings.

In this study, we address the following research question: is the lake island of Mörbisch in Lake Neusiedl, 1.8 km from the mainland and 2.0 km away from the next apiary, suitable for the establishment of an island mating station? We investigated mating reliability by examining the offspring of cd queens, with pure cd offspring indicating safe mating. As drone congregation areas are supporting local mating on the island, we provide drone colonies and additionally screen the island for drone congregation areas before and after the introduction of the drone colonies.

## 2 Material and methods

### 2.1 Mating reliability

Test bees were representatives of *Apis mellifera ligustica*, which have the recessive cordovan trait. The queens of the drone colonies were acquired in 2022 from the apiary Apis Johann Mädl (Mönchhof, Burgenland, AT) as 20 virgin queens and freely mated in Biedermannsdorf (Lower Austria, AT) between 1 and 13 May 2022. They are daughters of an instrumentally inseminated cordovan mother (further cd = cordovan, wt = wild type). As pure cd queens, they provide the cd drones needed for the experiment. The queens were taken out of their mating boxes on 13 May 2022 and put into the prepared queenless colonies on the island.

#### 2.1.1 Preparing the drone colonies

9 d before the planned introduction of the cd queens as drone suppliers (on 4 May 2022), the drone colonies at one of the authors' bee yards in Biedermannsdorf were prepared as follows: strong colonies of 10 to 12 Dadant combs were opened, and the entire combs with resident workers, drones, and queen were pushed into the hive. The combs, now with no bees sitting on them, were hung in six-comb boxes; to each box we added four brood combs, one supply comb (pollen and honey), and one empty drone comb brought from the home apiary (see below “Drone comb”). The remaining original brood box now contained one or two surplus combs with the entire bee mass of this colony. This was refilled with comb foundations from the home apiary. A queen excluder was placed on top of the brood box, allowing only the worker bees to pass through but neither drones nor the queen.

Above the queen excluder we put the two 6-comb boxes empty of bees, but filled with the combs of this colony. The two smaller colonies created from one big colony were left as one unity for 9 d until the arrival of the queens. The colonies were not moved or manipulated during this period. In the upper parts, excluding the queen and the drones, the brood was cared for by the workers, and food (nectar and pollen) was stored. On 13 May 2022 in the morning, as there is usually no drone flight at this time, we inspected the top boxes: all of the brood in them was capped. We removed and destroyed all capped drone brood cells. After this procedure, the boxes were placed on a closed-bottom board. The 14 queenless colonies were immediately moved to the island afterwards. After a short acclimatization phase, the colonies were given one cd queen each in a queen cage with candy. On 25 May 2022, the egg laying of queens was controlled, and the age of present brood suggested that queens began to lay eggs within 2 d of the introduction to colonies.

The colonies were brought to the island, containing only worker bees and sealed worker brood and no queens or drones. The four brood combs provided the hives with sufficient young bees, and for better care of the drone brood, which has a particularly high need for pollen during its rearing, 100–200 g protein dough (made of soy flour and sugar) was fed to the bees. There are a few small black locust trees (*Robinia pseudoacacia*) on the island, which were flowering at the time. As a result, many combs, especially the drone comb, were filled with fresh nectar and thus hindered the queen's egg laying. The drone combs of some hives therefore had to be replaced by new ones. On 30 May 2022, it was found that at least 8/14 queens had laid eggs all over the drone comb and another 3 had partially started or were in the process of doing so. Apparently, three colonies had lost the queen and were moved off the island. As the egg laying by the queens into the drone combs was completed between 16 and 30 May 2022 and drones require between 38 and 45 d from egg to sexual maturity (24 d of development in the cell and a further 14 to 21 d for sexual maturity), the highest number of sexually mature drones would have been around 12 July 2022 on the island. This date was scheduled as the date the virgin cd queen bees are brought to the island for mating.

#### 2.1.2 Drone comb

An already built drone comb was given to each drone colony (half of a Dadant comb) with a comb surface of 640 cm^2^, which, with an average size of the drone cells of 6.3–6.9 mm, results in about 1000 drone cells per comb side. Thus, about 2000 drones hatch from a drone comb from both sides. However, since quite a few drones are lost through the time of sexual maturity, we estimate there being approximately 500–1000 drones per drone colony, which results in 6000–10 000 mature drones in the remaining 11 drone colonies at the time of our experiments on the island.

#### 2.1.3 Setup of the drone colonies

The hives were set up on the premises of Mörbischer Tourismus BetriebsgesmbH (47°45^′^17.0^′′^ N, 16°41^′^35.4^′′^ E) in the western part of the island, near the swamp basin, which is sheltered from the wind in the reeds. The hive entrances were aligned to the east, i.e. away from the mainland (Fig. 2, A).

#### 2.1.4 Mating nucs

The mating nucs were brought to the island without any drones. The Swibi mating box (made by Swienty, Denmark) is easily transportable and provides space for the required number of bees to care for the queen and food.

#### 2.1.5 Virgin queen bees for mating

The virgin cd queens used for the experiment were provided by the apiary of Johann Mädl and were daughters of an instrumentally inseminated homozygous cordovan queen bee. In order to avoid inbreeding and the associated brood gaps due to the emergence of diploid drones (Mackensen, 1950), the cd drones of the drone colonies were not allowed to be too closely related to the virgin cd queens. We obtained different lines, with all bees having the cordovan trait.

#### 2.1.6 Arrangement of mating nucs

On 11 July 2022, 17 Swibi mating boxes with virgin cd queens were brought to the island and set up for their mating flights. They were placed about 300 m away from the drone colonies (Fig. 2, B). Originally, a second site was planned on the island, but it could not be used because people would have felt disturbed by the bees. 2 weeks later, on 25 July 2022, the mating nucs, together with the drone colonies, were transported from the island to the home apiary.

#### 2.1.7 Control group 1 – positive control

In order to prove that a mating of the cd drones with the cd queens on the island would produce cd workers exclusively, four cd queens were instrumentally inseminated by Thomas Sprenger with sperm from the cd drones out of the colonies on the lake island on 27 July 2022. The queen bees were 12–13 d old, were narcotized twice with CO_2_, and inseminated with 12 µ L semen from 10 to 15 drones. The inseminated queens were all laying fertilized eggs on 3 August 2022.

#### 2.1.8 Control group 2 – negative control

To prove that cd workers were sired by cd drones exclusively and no cd drones were in the surroundings of the lake, virgin cd queens were brought to the mating station Illmitz. It is located on the other side of the lake, opposite the lake island of Mörbisch. Of the six queens brought there on 17 July 2022, five mated and laid eggs (verified on 31 July 2022).

#### 2.1.9 Evaluation of the island offspring

After the mating nucs were brought from the island to the home apiary on 25 July 2022, they were fed with candy dough, and the queens had their wings clipped in order to guarantee that they would not fly out later and thus prevent them from mating outside the island. All the mating nucs were numbered at the home apiary to differentiate them.

On 1 August 2022, a brood comb with a very large amount of capped brood was taken out, the bees sitting on it were removed, and the comb was placed in a prepared transparent plastic box. To prevent it from drying out, it was lightly moistened with water. Most of the combs contained enough food to keep the hatching young bees well fed until they were counted. A few were given a ball of food dough to feed them during the short stay in the box.

**Figure 1:**
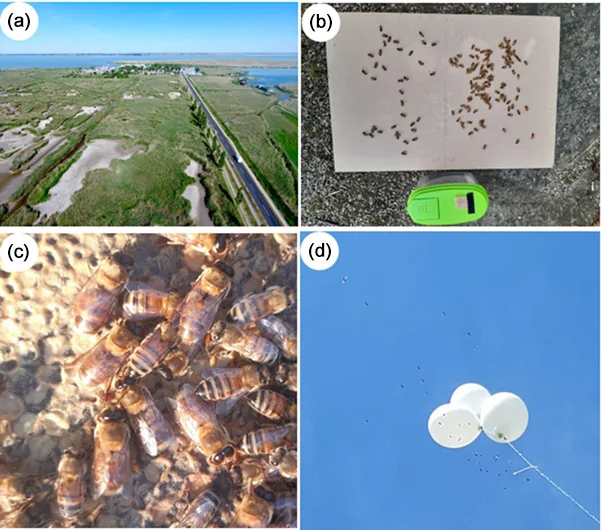
**(a)** View of the island from the middle of the dam. **(b)** Narcotized bees divided into wt/cd workers. **(c)** Cordovan drones with entirely brown abdomen, scutellum, legs, and antennae and regular wt worker bees. **(d)** Drones gathering around balloons and attractant at drone congregation area in Breitenfurt (method validation).

To count the cd/wt shares in the offspring of the island-mated queens, all the bees in the boxes were narcotized with CO_2_. The narcosis lasts about 3 to 4 min, which is just enough time to sort the bees and photograph the results. The narcotized bees were tipped onto a labelled pad, and the cd workers were separated from the wt workers (Fig. 1b). After sorting, both groups were photographed with the number of the mating unit, and later, the total number of bees and the proportion of cd bees were noted. The bees were then returned to their mating unit.

### 2.2 Drone congregation areas on the island

#### 2.2.1 Balloon method (16 May 2022)

In order to attract drones, first attempts were made with helium balloons. Four to five balloons were filled with helium, the balloons were tied together, and the string had metres marked in increments of 5 m. We used the attractant queen pheromone BeeBoost with QMP (Mio's Bienenwelt). The green pheromone stick was attached to a 3 cm long black straw 1 m below the balloons. This pheromone mimics the scent of a queen and increases attraction to drones. The black straw served as a visual stimulus to draw the drones' attention to the preparation. With this construction, the drone search on the island started. For the experiment, locations were mainly selected according to literature recommendations. In addition, particularly prominent places on the island were also investigated. In order to ensure an effective implementation of the balloon method, the weather conditions were carefully checked. The experiments took place on days with as little wind as possible and little cloud cover. On the one hand, this ensured a stable positioning of the balloons; on the other hand, the visibility of the drones could have been be maximized.

#### 2.2.2 Quadrocopter method (18 July 2022)

The quadrocopter model DJI Phantom 4 replaced the helium balloon. The pheromone stick was attached to a 3 cm long black straw and the quadrocopter with a string. GPS data from the quadrocopter gave precise information on the flight altitude, and the coordinates of the examined locations could be provided. These data enabled an accurate determination of the observed drone activities. The quadrocopter method proved to be an effective tool to collect data on drone activity. It offered greater mobility and flexibility compared to the air balloon method and enabled more precise measurements and observations.

#### 2.2.3 Maps

Maps were created to illustrate the examined locations. The DJI-GO 4 app recorded the flight routes of the quadrocopter and formed the data basis for the map layout. All flight routes were compared with the photos taken by the quadrocopter and the data from Google Maps, adjusted accordingly, and supplemented with the relevant coordinates. The graphic implementation of the cards was carried out with the professional design software Adobe Illustrator. The use of this software ensured an attractive and clear presentation of the results. The maps created offer a graphic overview of the examined locations and record the geographic distribution of drone activities.

**Figure 2:**
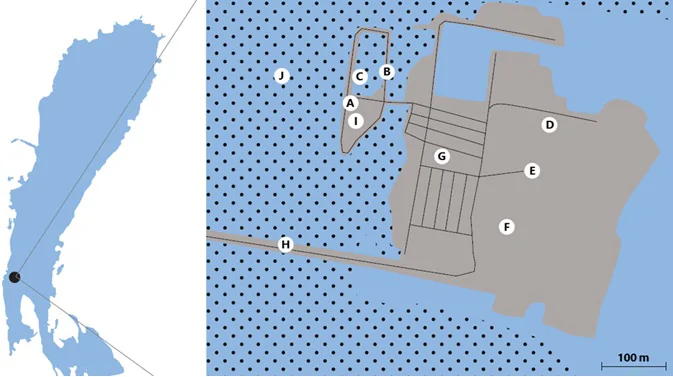
Overview of Lake Neusiedl with position of the island and locations at the island marked as A–J. Blue: water, dots: reed, grey: island.

## 3 Results

### 3.1 Mating reliability

Of the 17 queens placed on the island, 11 were laying eggs and the others were probably lost during their mating flights, leading to a mating flight returning rate of 59 %. The mating nucs were numbered after returning them to the home apiary, so the empty nucs were not further included into the statistics (Table 1). Queen number 11 only laid unfertilized eggs (drone eggs), the others (no. 1–10) produced healthy worker brood. All of the island-mated queens had different percentages of cd worker offspring. No. 9 was the only one that exclusively produced cd offspring; no. 4, on the other hand, did not mate with any cd drones at all considering its offspring. Most of the queen bees had over 50 % cd progeny; only no. 2 had 20 % cd workers (Table 1). Anyway, control group 1 (positive control) with the inseminated queens (No. 12–15) displayed cd workers exclusively. On the other end, control group 2 (negative control), the Illmitz mated queen bees, which only had access to wt drones, were producing 100 % wt workers (Table 1).

### 3.2 Drone congregations on the island

#### 3.2.1 Balloon method (16 May 2022)

The results of the first survey with the balloon method from 16 May 2022 are mapped in Fig. 2. Temperature and weather conditions were ideal at 27.5 °C and with a very light breeze only. The first place was the “swamp basin” (Fig. 2, C), about 20 m from the drone colonies. No drones were sighted between 5 and 12 m height. At 02:36 p.m., the balloon was released over the volleyball field (Fig. 2, D) next to the floating stage. There, too, no drones could be attracted over sandy ground at 10–17 m. No drone was attracted to the info point statue (Fig. 2, E), which is on asphalt/concrete. The same result followed for the paved right-rear parking lot 1 (Fig. 2, F) despite the demarcation with trees.

The parking lot 2 site (Fig. 2, G) has a slightly green lawn, and no drones were sighted here either. The dam between the mainland and the island (Fig. 2, H) was also free of drones. Overall, no drones could be found on the island until the drone colonies arrived.

To verify the effect of the pheromone and the construction, a negative control was carried out at two locations on the mainland. No drones could be attracted to the mainland of Mörbisch (47.7598220° N, 16.6595821° E). In Breitenfurt (48.135811° N, 16.198737° E), a drone congregation area was observed at 15:54 on 21 June 2022, which is proof of the effect of the pheromone balloon construction. The coordinates and names of the locations can be found in Table 2.

#### 3.2.2 Quadrocopter method (18 July 2022)

The result of the second survey on 18 July 2022 was significantly different (Table 3). Due to the presence of drone colonies on the island, drones could be attracted to the quadrocopter this time. With comparable weather conditions of 29 °C and mostly no wind, the start was made at parking lot 2 (Fig. 2, G). Two to three drones could be attracted here.

No drones could be attracted or observed at parking lot 1 (Fig. 2, F), at the info point statue (Fig. 2, E), and on the sea route (Fig. 2, H). At the swamp basin (Fig. 2, C), which was completely dry at the time of the survey, a large number of drones could be sighted with the quadrocopter. The number was estimated to be between 100 and 200 drones. Another observation showed that the drones gave up the pursuit as soon as the quadrocopter with the pheromone stick left the reeds and headed for the water surface (Fig. 2, J). When the quadrocopter flew over the land again, a handful of drones could be observed at the gravel pit (Fig. 2, I) a few metres from the swamp basin.

**Table 1:** Offspring evaluation of all laying queens of the experiment. The sum of each sample group is in bold.

Mating unit	Sample group	Location	Sample size	Number of	Percentage of
number			(number of emerged bees)	cd worker bees	cd workers
1	Experimental	Lake island	129	110	85 %
2	Experimental	Lake island	119	26	22 %
3	Experimental	Lake island	146	120	82 %
4	Experimental	Lake island	62	0	0 %
5	Experimental	Lake island	121	78	64 %
6	Experimental	Lake island	150	113	75 %
7	Experimental	Lake island	123	67	54 %
8	Experimental	Lake island	223	126	57 %
9	Experimental	Lake island	70	70	100 %
10	Experimental	Lake island	142	95	67 %
11	Experimental	Lake island	unfertilized	–	–
∑	**Experimental**	**Lake island**	**1285**	**805**	**63 %**
12	Control 1 pos.	Inseminated	106	106	100 %
13	Control 1 pos.	Inseminated	121	121	100 %
14	Control 1 pos.	Inseminated	103	103	100 %
15	Control 1 pos.	Inseminated	87	87	100 %
∑	**Control 1 pos.**	**Inseminated**	**417**	**417**	**100 %**
16	Control 2 neg.	Illmitz	116	0	0 %
17	Control 2 neg.	Illmitz	98	0	0 %
18	Control 2 neg.	Illmitz	124	0	0 %
19	Control 2 neg.	Illmitz	115	0	0 %
20	Control 2 neg.	Illmitz	109	0	0 %
∑	**Control 2 neg.**	**Illmitz**	**562**	**0**	**0 %**

**Table 2:** Investigated sites on 16 May 2022. Letters in parenthesis in location refer to Fig. 2.

Location	Coordinates	Date	Time	Height	Weather	Temp.	Under ground	Drones	Drone	Balloon/
	(° N, ° E)			(m)		(°C)		found	number	quadrocopter
Swamp (C)	47.7554202, 16.6929736	16 May 2022	14:23	5–12	windy	28	Swamp	–	0	Balloon
Volleyball field (D)	47.7541205, 16.6973214	16 May 2022	14:36	10–17	still	28	Sand	–	0	Balloon
Info point statue (E)	47.7537515, 16.6966489	16 May 2022	14:49	7–10	soft breeze	28	Asphalt	–	0	Balloon
Parking lot 1 (F)	47.7528866, 16.6964370	16.05.22	14:57	10–15	still	28	Asphalt/trees	–	0	Balloon
Parking lot 2 (G)	47.7533538, 16.6948383	16.05.22	15:05	10–20	still	28	Lawn	–	0	Balloon
Dam (H)	47.7535040, 16.6841015	16.05.22	15:17	10	soft breeze	28	Reed	–	0	Balloon
Mörbisch (mainland)	47.7598220, 16.6595821	16.05.22	15:38	10–20	still	28	Reed/lawn	–	0	Balloon
Breitenfurt(mainland, control)	48.135811, 16.198737	21.06.22	15:54	10–25	still	28	Meadow	+	100–200	Balloon

**Table 3:** Sites investigated on 18 July 2022. Letters in parentheses in location refer to Fig. 2.

Location	Coordinates	Date	Time	Height	Weather	Temp.	Under ground	Drones	Drone	Balloon/
	(° N, ° E)			(m)		(°C)		found	number	quadrocopter
Swamp (C)	47.7554202, 16.6929736	16 May 2022	16:04	12	still	29	Swamp	+	100-200	Quadrocopter
Volleyball field (D)	47.7541205, 16.6973214	16 May 2022	15:48	12	still	29	Sand	+	5–7	Quadrocopter
Info point statue (E)	47.7537515, 16.6966489	18.07.22	15:36	15	still	29	Asphalt	–	0	Quadrocopter
Parking lot 1 (F)	47.7528866, 16.6964370	18 July 2022	15:27	12–20	still	29	Asphalt/trees	–	0	Quadrocopter
Parking lot 2 (G)	47.7533538, 16.6948383	18 July 2022	15:13	12–20	still	29	Lawn	+	2–3	Quadrocopter
Dam (H)	47.7535040, 16.6841015	18 July 2022	16:52	15	still	29	Reed	–	0	Quadrocopter
Gravel pit (I)	47.754397, 16.693124	18 July 2022	16:29	12	soft breeze	29	Reed/gravel	+	5–7	Quadrocopter
Shore (J)	47.754933, 16.691794	18 July 2022	16:20	13	soft breeze	29	Reed/water	–	0	Quadrocopter

## 4 Discussion

The average percentage of cd bees found from queens mated on the investigated island is 63 %, which is within the range of reliability from the tests carried out by Maul (1972). Since the proportion of represented drones (fathers) in the existing workers is not homogeneous, the degree of mismating by means of number of drones cannot be determined exactly. However, studies of a queen over 3 years showed that the representation of drones as fathers of the worker bees is not homogeneous at all and there are numerous fathers that are over- or underrepresented (Brodschneider et al., 2012), so the degree of workers sired by wt drones versus cd drones cannot exactly be determined. To get more precise information about the number of drones the queen bees mated with and their proportion in the queens' progeny, a molecular paternity test would have been more suitable. Nevertheless, although the percentage of each drone's daughters cannot be determined exactly, the cd test still gives a good overview of the proportion of intended matings. According to Ruttner (1979), at least half of the queen bees from an island should have cd daughters exclusively to make it a useful mating station. If a queen's offspring is cordovan-coloured exclusively, the proportion of fathers loses its importance since the bees are all a product of the intended mating.

All mating stations have a potential for outside influences; pure matings are best achieved by instrumental insemination (Cobey, 2007). Genetic studies of the offspring of queens mated at the Carnica mating stations Gehlberg (Thuringia) and Hassberge (Bavaria) showed a proportion of mismatings between 20 % and 30 % depending on how many drone colonies were used there (Bieneninstitut Kirchhain, 2012). Although these results are better than those obtained on the lake Neusiedl island, it should be mentioned that in the case of the Gehlberg and Hassberge experiments, 20–50 and sometimes even more than 70 drone colonies were used, whereas in the current experiment, only 11 drone colonies were used.

The cordovan tests of Ruttner and Ruttner (1965) on the island Bauminsel in lake Neusiedl showed similar results to our investigation: very high losses among the queens and a similar percentage of cd matings. Similarly, our results suggest that all queens, except one, visited the mainland to mate with the wt drones there. We base this on drones even avoiding crossing small water surfaces (Ruttner and Ruttner, 1965; Neumann et al., 1999). However, we cannot completely rule this out, as the reed lowers the perception of the water surface, and the water level of the lake was at a low during the year of the experiment. Catching drones from the drone congregation area on the island would have helped secure this knowledge.

The methodology with homozygous cd queens was successfully validated, as shown with control group 1, via instrumental insemination. The mating of cd queens and cd drones results exclusively in cd offspring. Control group 2 (negative control) results prove that no cd drones were flying to the mainland across the lake or that none of this group's queen bees flew 6 km from Illmitz across the lake to mate with Mörbisch Island cd drones.

Increasing the number of drone colonies on the island could probably lead to equally good if not better results. However, according to currently available data, the minimum of 50 % intentional matings required by Ruttner (1979) cannot be achieved by all of the queens. The suitability as a mating station is therefore only limited depending on how it would be operated in the future. The hypothesis that with enough present drones on the island, the mating distance over water would be reduced can obviously not be confirmed, the mating biology of the honey bee aims at the highest possible genetic diversity in the offspring of a queen (Koeniger, 1986), which is why all queens except one have chosen the risky way to the mainland. This route most likely ended fatally for some of the queens, which would explain the high mating losses of 41 %. The flights to the mainland were not necessary for the queens due to the high presence of drones on the island. Still, queens flew to the mainland, taking a high risk. Again, the reed and the low water level might have reduced the possible barrier for the queens. The results showing mixed matings of queens with cd and wt drones (queens from mating units 1–3, 5–8, and 10) suggest that queens performed several mating flights (Heidinger et al., 2014).

There might be a concern about a lower fitness of the cd drones causing a higher influence of the wt drones (Berg et al., 1997), but according to Ruttner and Ruttner (1965), there is no significant difference between cordovan and wild-type drones. Anyway, the lower success rate in mating of cd drones in the experiments of Berg et al. (1997) may be explained by the use of a highly inbred stock (Moritz, 1981) because Peer (1957) used unrelated cordovan lines and could not confirm a lower fitness of any manifestation in cd drones. For the current experiment, drone colonies had only slight inbreeding and did not appear any different to regular wt drones. According to Koeniger et al. (1989), *A. m. carnica* and *A. m. ligustica* (used for this investigation) mate at different heights. This could have affected the results positively because the wt drones would have had a disadvantage in this situation. However, local beekeepers in Mörbisch and around Mörbisch are working with *A. m. carnica* as well as *A. m. ligustica* and the Buckfast strain (related to *A. m. ligustica*), so there would have been no advantages for the *A. m. ligustica* cd drones due to their mating altitude in this situation.

To prove that drone congregation areas only set up on the island when the cd drone colonies were there, we had to test several sites on the island before the arrival of the drone colonies. During the test in May, where no colonies were on the island, no drones could be found, whereas in July, a drone congregation area could be verified above the swamp near the cd drone colonies. Böttcher (1975) showed that if there are drones present, they can be attracted everywhere in small numbers, whereas drone accumulations up to 100 and more individuals can only be found at certain drone congregation areas. Therefore, the swamp site appears to be such a drone congregation area. Whether this drone congregation area only contained cd drones or wt drones as well could not be detected. It is therefore possible, but unlikely, that wt drones joined the drone congregation area on the dry swamp after it was built up by the cd drones from the colonies on the island.

To conclude, an island surrounded by reed, as Mörbisch Island investigated here, is not suitable for installing a mating station for breeding programmes. Furthermore, we follow Klatt (1929), Klöpping (1993), and Neumann et al. (1999) in assuming that queens crossed the water surface even though enough sexual mates were available on the island.

## Data Availability

The authors confirm that the data supporting the findings of this study are available in Tables 1, 2, and 3.
